# Reducing sitting at work: process evaluation of the SMArT Work (Stand More At Work) intervention

**DOI:** 10.1186/s13063-020-04300-7

**Published:** 2020-05-13

**Authors:** Stuart J. H. Biddle, Sophie E. O’Connell, Melanie J. Davies, David Dunstan, Charlotte L. Edwardson, Dale W. Esliger, Laura J. Gray, Thomas Yates, Fehmidah Munir

**Affiliations:** 1 Centre for Health Research, University of Southern Queensland, Springfield,, QLD 4300 Australia; 2 Leicester Diabetes Centre, University Hospitals of Leicester, Leicester General Hospital, Leicester, LE5 4PW UK; 3 Diabetes Research Centre, University of Leicester, Leicester General Hospital, Leicester, LE5 4PW UK; 4 NIHR Leicester Biomedical Research Centre, Leicester, UK; 5 Baker Heart and Diabetes Institute, Melbourne, VIC Australia; 6 Mary MacKillop Institute for Health Research, The Australian Catholic University, Melbourne, VIC Australia; 7 School of Sport, Exercise and Health Sciences, Loughborough University, Loughborough, UK; 8 Department of Health Sciences, University of Leicester, Leicester, UK

**Keywords:** Workplace, Behaviour change, Sedentary behaviour, Sit–stand

## Abstract

**Background:**

Office-based workers accumulate high amounts of sitting time. Stand More At Work (SMArT Work) aimed to reduce occupational sitting time and a cluster randomised controlled trial demonstrated it was successful in achieving this aim. The purpose of this paper is to present the process evaluation of the SMArT Work intervention.

**Methods:**

Questionnaire data were collected from intervention participants at 6 months (*n* = 58) and 12 months (*n* = 55). Questionnaires sought feedback on the different components of the intervention (education, height-adjustable desk, Darma cushion, behaviour feedback, progress chats (coaching) with research team, action planning/goal setting diary) and experiences of evaluation measures. Control participants (*n* = 37) were asked via questionnaire at 12-month follow-up about the impact of the study on their behaviour and any lifestyle changes made during the study. Participants from both arms were invited to focus groups to gain a deeper understanding of their experiences on completion of 12-month follow-up.

**Results:**

Focus group and questionnaire data showed a positive attitude towards the height-adjustable workstation with a high proportion of participants using it every day (62%). Most participants (92%) felt the education seminar increased their awareness of the health consequences of too much sitting and motivated them to change their behaviour. Receiving feedback on their sitting time and support from the research team also encouraged behaviour change. The Darma cushion and action planning/goal setting diary were seen to be less helpful for behaviour change. Benefits experienced included fewer aches and pains, improved cognitive functioning, increased productivity, more energy and positive feelings about general health.

**Conclusions:**

Key elements of the programme identified as facilitating behaviour change were the educational seminar, the height-adjustable workstation, behavioural feedback and regular contact with research staff through regular progress chats.

**Trial registration:**

ISRCTN: ISRCTN10967042. Registered on 2 February 2015.

## Background

High levels of sedentary behaviour (sitting with low energy expenditure) have been shown to be detrimentally associated with a number of physical and mental health outcomes [1–3]. For example, the US 2018 Physical Activity Guidelines Committee concluded that there was ‘strong evidence for a direct association between greater amounts of sedentary behavior and higher risk of mortality from all-causes and CVD, and for higher risk of type 2 diabetes and CVD’ [4]. Moreover, with trends towards greater sitting in the workplace [5], office workers have been shown to engage in high levels of sitting [6]. The Stand More At Work (SMArT Work) programme was an intervention tested in desk-based employees of an English East Midlands National Health Service (NHS) Trust [7–9]. A full study protocol is published [7] but, in brief, groups of desk-based staff within the same offices were randomised to either an intervention or control condition. The intervention participants received a multi-component intervention designed to reduce workplace sitting. Those in the control office clusters continued with their usual practice.

The logic model of the randomised controlled trial (see [8]) stated that the intervention was grounded in several behaviour change theories and implemented through the intervention functions of the Behaviour Change Wheel. These included organisational, environmental, and individual and group functions, and informed elements of the process evaluation.

Results at 12 months showed favourable changes in the intervention group relative to controls for occupational sitting time, prolonged sitting, standing time, some musculoskeletal issues, various occupational measures (job performance, work engagement, occupational fatigue, sickness presenteeism), and quality of life [8].

Process evaluation provides information concerning implementation (e.g. fidelity, reach), possible reasons for outcomes (mechanisms of impact, such as participant responses and mediators), and contextual factors shaping intervention outcomes [10]. Given the multi-component nature of this intervention, it was important to understand how participants viewed each component as well as the intervention overall. Therefore, to better understand how the SMArT Work intervention operated and was perceived by participants, we undertook several process evaluation assessments with the intervention participants (questionnaires at 6-month and 12-month follow-up and focus groups at 12-month follow-up) and control participants (questionnaire and focus groups at 12-month follow-up only).

## Method

Ethical approval was obtained from Loughborough University, and Research and Innovation approval was obtained from the University Hospitals of Leicester NHS Trust (EDGE ID 34571). All individual participants provided informed consent on entering the study.

A sequential exploratory mixed-methods approach was adopted with data collected from both intervention and control participants using questionnaires and focus groups. Table 1 shows the main methods and data collected.

**Table 1:** Process evaluation methods and data collected for intervention and control participants

Focus	Intervention participants			Control participants	
	6-month questionnaire (*n* = 58, 88% RR)	12-month questionnaire (*n* = 55, 87% RR)	Focus group (*n* = 29, 46% RR)	12-month questionnaire (*n* = 37, 80% RR)	Focus group (*n* = 5, 11% RR)
Height-adjustable workstation					
Use	√	√	**√**		
Usability	**√**	**√**	**√**		
Experiences	**√**	**√**	**√**		
Perceptions	**√**	**√**	**√**		
Strategies for use	**√**	**√**	**√**		
External support for use			**√**		
Education seminar					
Perceptions of content	√		√		
Increased awareness and motivation	√				
Key messages	√		√		
Impact on behaviour			√		
Wider dissemination					
Sitting diary (for goal setting/self-monitoring behaviour)					
Use	√	√			
Usefulness	√	√			
Improvements	√	√			
Sitting behaviour feedback					
Use	√	√			
Usefulness	√	√			
Increased motivation	√	√			
Assisted with goal setting	√	√			
Darma cushion					
Use	√	√			
Usefulness	√	√			
Usability of device	√	√			
Ease of use	√	√			
Facilitated behaviour change	√	√			
Alternative support for self-monitoring and/or prompt					
Use of other self-monitoring or prompt tools	√	√			
Educational leaflet					
Use	√				
Usefulness	√				
Perceptions of content	√				
Progress chats (coaching) with research team					
Usefulness		√			
Perceptions		√			
Motivations for remaining in study					√
Workplace managerial support			√		√
Strategies used to change sitting behaviour (desk, goal setting, prompting, education)			√		
Behaviour changes resulting from participating in study			√	√	
Motivators for behaviour change			√		
Facilitators to behaviour change			√		
Impact of colleagues on behaviour			√		
Reducing sitting outside of work			√		
Benefits of reducing sitting and negative/adverse events			√		
Behaviour change maintenance			√		
Wider policy changes at work			√		
Other lifestyle changes	√	√		√	
Impact of measurement sessions on behaviour			√	√	√

*RR Response Rate*

### Questionnaires

A mix of open-ended, forced choice, and Likert scaled questions were used in the questionnaire. Intervention participants completed questionnaires at 6 months (*n* = 58, 88% of intervention participants still in the study; 74% female; body mass index 25.8 ± 5.0 kg/m^2^; age 42.4 ± 11.3 years) and 12 months (*n* = 55, 87% of intervention participants still in the study; 71% female; body mass index 26.0 ± 5.3 kg/m^2^; age 43.0 ± 10.8 years). Questionnaires for the intervention participants sought feedback on the following main elements of the intervention (see Table 1):

- Educational seminar and leaflet; a 30-min group educational seminar concerning the health consequences of sitting and the benefits of reducing or breaking up sitting and a leaflet to reinforce the key messages. Feedback was sought at 6 months only due to the one-off nature of the seminar.
- Feedback on their own sitting, standing and stepping generated from the activPAL monitor.
- Height-adjustable workstation; participants were given the choice of two models (full electric desk or a choice of two sizes of an adjustable platform which sat on their existing desk).
- Use of a sitting time diary, including action planning and goal setting.
- Use of the Darma cushion, a cushion placed on office chair which connected to a smart phone via Bluetooth. Real-time feedback on sitting is provided along with a vibration prompt to break up sitting regularly.
- Brief coaching sessions (‘progress chats’); these took place every few months throughout the intervention. Feedback was sought at 12 months only.

Questionnaires for the control participants (*n* = 37, 80% of control participants still in the study at 12 months) sought feedback on the impact of study measurement sessions and receiving health results. All participants were asked whether other lifestyle changes had been made during the study that might impact on the results, such as moving house or joining a gym.

### Focus groups

Participants were invited to attend a focus group following completion of 12-month follow-up. Focus groups were led by one researcher (SEO’C) and were semi-structured with a focus group guide devised by the wider research team. Seven focus groups, lasting between 40 and 64 min, took place with 29 intervention participants (46% of intervention participants still in the study at 12 months; 72% female; body mass index 26.1 ± 5.6 kg/m^2^; age 41.1 ± 12.2 years), representing 16 intervention clusters (84%). The focus group discussion topic guide gathered responses concerning: 1) experiences of each intervention component; 2) the facilitators to take part in the study, and if and how their behaviour changed; 3) insight into the strategies they used to change their behaviour and their experiences of reducing sitting behaviour; 4) benefits and/or negative experiences of the intervention and discussions around sustaining new behaviour; and 5) how the messages of SMArT Work could be rolled out (see Table 1).

Two brief focus groups, lasting between 8 and 12 min, were held with five control group participants (11% of control participants still in the study at 12 months; 80% female; body mass index 25.8 ± 3.7; age 50.4 ± 14.3 years) representing four clusters (25%). Questions aimed to gather insight into why they took part, what motivated them to stay in the study once allocated to the control arm, whether they felt supported through the project by their manager and how the measurement feedback impacted them in any way.

### Data analysis

Forced choice and Likert scaled questionnaire items were analysed with frequency counts or means and standard deviations using IBM SPSS v25. Open-ended responses from the questionnaire were grouped into coherent themes (by SJHB) using template analysis [11]. All statements were entered into MindGenius (v6) software and grouped by themes and sub-themes. Audio recordings from the focus groups were transcribed verbatim. A combined deductive and inductive approach was used to analyse the data using template analysis. The first stage of this template analysis was to define the themes relevant to the discussion topics outlined above. Two members of the research team (SEO’C and FM) independently applied these themes to the focus group dataset to develop a template depicting the salient themes. Data that did not fit the initial template, but were relevant to the research aims, were coded and the themes were continuously modified as the data were interpreted, until a final template of five intervention focus group main themes were created and one control group main theme was created (see Table 2). There was agreement between the two researchers on the template themes identified and an additional theme around the incidental culture of standing at work. Results were triangulated to integrate findings from focus groups and questionnaires.

**Table 2:** Template analysis: themes and sub-themes from intervention and control focus groups

Level 1 main theme	Level 2 sub-theme	Level 3 sub-theme
Intervention group (*n* = 29)		
Attitude and behaviour change regarding reducing sitting at work	Factors that promote reductions in sitting and habit formation	Seminar
		Feedback on physiological, anthropometric and activPAL feedback
		Prompts
		Social influence
		Length of intervention study
	Factors that did not help with reductions in sitting	Diary
		Cushion
	Standing further reinforces other attitude and/or behaviour change	Other behaviour changes at work
		Sitting less at work
		Other external impacts
Creating an incidental socio-cultural environment of standing at work	None	None
Perceptions of the benefits of standing	Health benefits	Reduction in musculoskeletal problems
	Work-related benefits	Productivity
		Changes in work style
		Interaction with colleagues
Barriers to behaviour change	Lack of motivation	Goal setting
	Aspect of the job	None
Wider policy changes at work	Organisational-wide communication	None
	Mandatory training	
Control group (*n* = 5)		
Motivations to stay in the study	Manager support	None
	Interest in research	
	Low time commitment	
	Feedback from health measures	

## Results

**Fig. 1:**
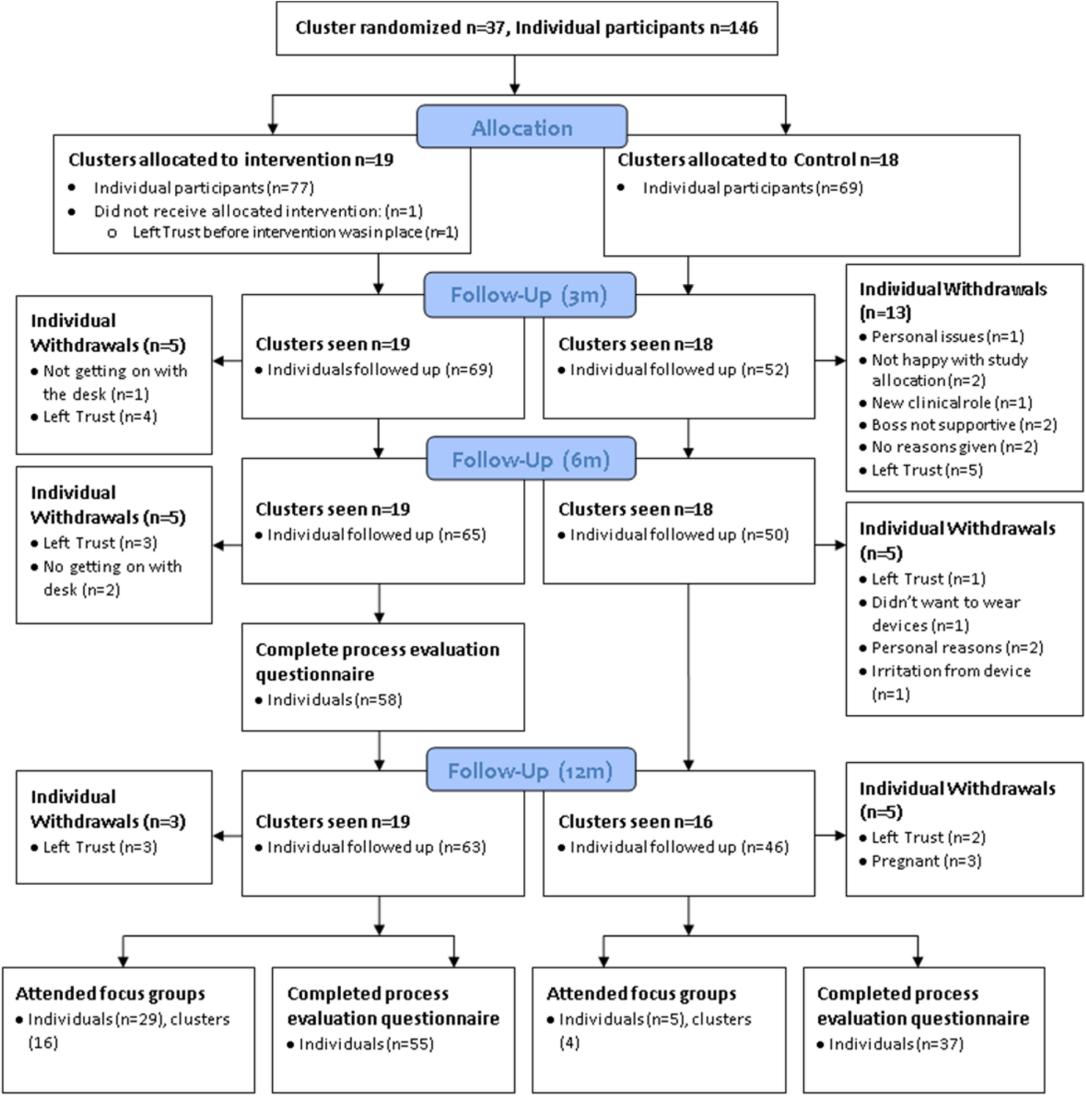
Flow of participants through the randomised controlled trial

Results are presented mainly according to intervention components with quantitative and qualitative data from the questionnaires integrated throughout the results together with the themes identified from the focus groups (shown in Table 2.) Figure 1 shows the flow of participants through the randomised controlled trial.

### Educational seminar and leaflet

Only seven intervention participants (14%) did not attend the seminar face-to-face and were sent an audio recorded presentation instead. From those who had attended the seminar, questionnaire data (Table 3) showed strong agreement between the respondents that the seminar was delivered at an appropriate level (94% agreed or strongly agreed) and was an appropriate length (95% agreed/strongly agreed). Importantly, 93% felt that the seminar increased their awareness of the health consequences of too much sitting and 95% felt it motivated them to make a change to the amount of time they spend sitting.

**Table 3:** Responses concerning the education seminar intervention participants received

Statement	6 months	
	Agree (*n*/%)	Strongly agree (*n*/%)
The education seminar was pitched at the appropriate level	14/26%	36/68%
The education seminar was the appropriate length	21/40%	29/55%
The education seminar increased my awareness of the health consequences of too much sitting	13/25%	36/68%
The education seminar motivated me to make a change to the time that I spend sitting	20/38%	30/57%

Open-ended questionnaire responses were requested concerning understanding the key seminar messages. Of the 58 respondents, 48 provided comments, with three main questionnaire themes emerging:

- standing and breaking up sitting is desirable to prolonged sitting
- excessive sitting is bad for your health
- exercise may not offset the detrimental effects of prolonged sitting.

In the focus groups, some participants discussed how knowledge learned from the seminar had a powerful impact on their understanding and attitude toward excessive sitting at work [level 1 main theme—attitude and behaviour change regarding reducing sitting at work].

‘I’m quite aware of the effects of sitting all day but I think it’s not until you sit there and actually listen to all the information that you think okay yes this can actually have a huge impact’ (Intervention participant, focus group 4).

Of the questionnaire respondents, 89% said that they had read the educational leaflet provided. The content was seen as appropriate (88% rating agree/strongly agree). Most (88%) felt that the leaflet increased their awareness of the health consequences of too much sitting and 82% said it motivated them to make a change to the amount of time they spent sitting.

### Height-adjustable workstation

Although participants were given a choice of desk, they chose roughly evenly between the two models (60% chose a Varidesk platform and 40% the electric workstation). The purchasing and delivery of the workstations took longer than planned and so many of the participants would have only had the workstation installed for ~1 month prior to the 3-month follow-up visit. Table 4 presents the quantitative feedback on the workstation. At 6 months, all those responding said that they had used the workstation at least once during the first 6 months, with all but three respondents reporting using the workstation at least a few times per week (33%) or everyday (67%). In the first month of installation, 67% used it every day, with similar rates between desk type. The majority of participants did not find their workstation obtrusive to completing work tasks at 6 months (69%) and 12 months (67%). Questionnaire data showed that respondents were not self-conscious when using the workstations (84% and 88% at 6 months and 12 months, respectively), nor did they think their colleagues minded when the workstation was being used (98% at 6 months and 99% at 12 months). These data were supported in the focus groups with participants highlighting that their non-participant colleagues did not impact on their use of the height-adjustable workstation [level 3 sub-theme—social influence].

‘The people who didn’t have the desks, they didn’t say anything or, like it wasn’t awkward to stand up at any point in the office or anything like that, it was fine’ (Intervention participant, focus group 3).

Questionnaire respondents were asked how they scheduled the use of their workstations, such as specifying certain times or for particular tasks. At 6 months, 44% reported scheduling often or very often, with a slight drop to 36% at 12 months. The mixed responses were reinforced by the focus groups with some people stating that they had set times/periods when they used their desk, such as first thing in the morning or in the afternoon, but for some people it was used more randomly or when their body felt like it needed a change in posture [level 1 main theme—perceptions of the benefits of standing; level 3 sub-theme—feedback and prompts].

‘I come in the morning and I automatically put my desk up. I’ll stand until I’m starting to feel … I’m not standing properly. So I’ll just sit down, but then when I’m taking phone calls or particular slots for e-mails I then stand’ (Intervention participant, focus group 4).

‘My back lets me know when I need to stand up’ (Intervention participant, focus group 1).

Regarding perceptions of whether the workstations helped the participants reduce their sitting time, 95% and 94% of questionnaire respondents reported agreement at 6 months and 12 months, respectively. Agreement was high and broadly similar at 12 months between the electronic workstation (100%) and the Varidesk (92%). However, it was also evident from the focus group discussions that some people stood for prolonged periods at their desk despite regular posture change being recommended [level 3 sub-theme—sitting less at work; level 1 main theme—perceptions of the benefits of standing].

‘I had a period of time when I’ve come in every morning and lifted it up and I’d stand at it until I’d had enough. You know sometimes it was two hours and then I’d put it down and think I’m done for the day now’ (Intervention participant, focus group 1).

‘I stand for ages … but I like that, it feels really good for me’ (Intervention participant, focus group 3).

At 6 months and 12 months, questionnaire respondents were asked to write comments concerning what was positive about using their workstation. From 69 and 79 statements provided at 6 months and 12 months, respectively, six main themes emerged:

- musculoskeletal and posture: better posture and fewer aches and pain, especially in the neck, shoulders and back
- mental benefits: better cognitive functioning and work productivity (e.g. ‘feel more productive’, ‘allowed me to concentrate and focus’), enhanced mood (‘feel my mood has improved’), and improved feelings of energy and alertness (e.g. ‘re-energises me’, ‘felt more alert throughout the day’)
- more movement: ‘I am more likely to move about the office if standing’
- general health benefits: ‘feel more positive about my health’
- social norms and benefits: ‘more are willing to stand if I am standing’
- increased choice: ‘gives me the option’

**Table 4:** Responses concerning the height-adjustable workstation

	6 months (*n*/%)	12 months (*n*/%)
Have you used the workstation in the last 6 months? (Yes)	56/100%	54/98%
In the first month of receiving the height-adjustable workstation how often did you use it (i.e. moved it from a sitting to standing position or vice versa)?		
Everyday	39/67%	–
A few times a week	18/31%	–
Once a week	1/2%	–
Infrequently	0/0%	–
In the past 6 months how often have you used the height-adjustable workstation (i.e. moved it from a sitting to standing position or vice versa)?		
Everyday	36/67%	39/72%
A few times a week	19/33%	13/24%
Once a week	1/2%	0/0%
Infrequently	2/3%	2/4%
When I use my desk (i.e. moved it from a sitting to standing position or vice versa) I schedule specific times to use it (e.g. every hour for a certain length of time, just in the afternoon, or during a specific task such as reading emails) to stand up		
Very often	9/15%	10/18%
Often	17/29%	10/18%
Sometimes	19/33%	24/44%
Rarely	7/12%	9/16%
Never	6/10%	2/4%
When I use the height-adjustable workstation to stand I feel self-conscious		
Strongly disagree	28/48%	30/55%
Disagree	21/36%	18/33%
In the past 6 months my office colleagues have not minded when I use the height-adjustable workstation to stand and work		
Strongly agree	45/82%	41/79%
Agree	9/16%	11/20%
In the past 6 months the height-adjustable workstation has helped me to reduce the amount of time I spend sitting at work		
Strongly agree	37/64%	37/67%
Agree	18/31%	15/27%
How obtrusive has the height-adjustable workstation been to your daily activities at work (i.e. does it prevent you from working efficiently and effective)?		
Very obtrusive	1/2%	1/2%
Mildly obtrusive	13/22%	11/20%
Neither obtrusive nor unobtrusive	5/9%	6/11%
Mostly unobtrusive	13/24%	9/16%
Completely unobtrusive	26/45%	28/51%

Additional themes concerned ‘comfort and utility’ at 6 months (e.g. ‘more comfortable standing’, ‘easy to use’) and ‘ergonomics’ at 12 months (e.g. ‘more natural eye line to the screen’).

Musculoskeletal outcomes were also reflected in the data from the focus groups [level 3 sub-theme—reduction in musculoskeletal problems]:

‘Prior to this study, I did have problems with my shoulder and I’ve found standing does alleviate that because when you’re with your mouse like that of at the keyboard, and now standing, it’s different, your hands are lower, and I’ve not had a problem with my shoulder since the study’ (Intervention participant, focus group 6).

Many focus groups participants described how standing up during tasks led to increased productivity and confidence [level 3 sub-theme—productivity]:

‘I feel I work better, I work faster when I am standing up, to be honest, when you are sitting down, you are sort of just there’ (Intervention participant, focus group 6).

‘Busy and stressful before, I just had hundreds of emails hitting me, phone calls, doctors coming in, there was so much, but even then, I still stood up during then, I thought, actually, it made me feel like that different mindset … I felt more confident standing up … I felt I could deal with things’ (Intervention participant, focus group 3).

As the randomised controlled trial was a cluster design, where groups of people within the same office group were randomised to the same group, if one person was standing up (at their desk or elsewhere) this would often remind other colleagues to also stand up. Therefore, a knock-on effect of colleagues standing regularly was evident, thus creating a culture shift, as illustrated by these comments from the focus groups [level 1 main theme—creating an incidental socio-cultural environment of standing at work]:

‘In the environment when there’s lots of people standing up, you know, one person stands up, you know, then it’s oh yes, I need to stand up, too’ (Intervention participant, focus group 6).

‘I think it’s like a culture of, like, in an office where everybody is doing the same thing then it’s almost like an instant reminder, you know, maybe I should be standing at the same time and, you know, where it’s like a team sort of thing you get into sort of, like, a routine where everybody will be standing at some point, you know, during the day, and encourages it’ (Intervention participant, focus group 6).

Questionnaire respondents were also asked to write comments concerning what was negative about using the workstation at both 6 months and 12 months. The main issue that emerged concerned the lack of space on the desk and concern about papers and files falling off, and a lack of space for handling multiple papers. These comments were exclusively in reference to the Varidesk. This was also highlighted in the focus groups by some participants.

‘Initially it was the lack of space. Because it was a two-tiered system, when you did stand up there was not much space to put your paperwork on’ (Intervention participant, focus group 2).

### Darma Cushion

The questionnaire findings (Table 5) showed the use of the Darma cushion and associated app was moderate at 6 months; 55% reported using it since it was given to them and few planned to use it in the future. Assessing over the past 6 months, users of the Darma cushion reported varied responses, with 39% reporting infrequent use, while 36% reported daily use. Only a small percentage (15%) of participants viewed their feedback on the app frequently. Only 11 (20%) reported using the cushion in the last 6 months at the 12-month time point, with 18% reporting infrequent use, 36% using it ‘a few times per week’, and 46% reporting daily use. The use of the Darma cushion was initially reasonable with 68% reporting daily use in the first month. The cushion vibration function was used by 62% and 46% of those that reported using the cushion in the past 6 months at the 6-month and 12-month time points, respectively, with most (87% and 70% at 6 months and 12 months, respectively) reporting it to be useful.

**Table 5:** Responses concerning the Darma cushion

	6 months (*n*/%)	12 months (*n*/%)
Have you used the Darma cushion since we gave it to you?		
Yes	32/55%	11/20%
No	26/45%	43/80%
In the past 6 months how often have you used the Darma cushion?		
Every day	12/36%	5/46%
Few times/week	8/24%	4/36%
Once/week	0/0%	0/0%
Infrequently	13/39%	2/18%
In the past 6 months how often have you viewed your sitting time feedback on the app?		
> once/day	2/6%	1/8%
Once/day	3/9%	0/0%
Few times/week	7/21%	3/25%
Once/week	0/0%	0/0%
Infrequently	22/65%	8/67%
In the first month of receiving the cushion how often did you use it?		
> once/day	23/68%	–
Once/day	5/15%	–
Few times/week	0/0%	–
Once/week	5/15%	–
Infrequently	1/3%	–
In the first month of receiving the cushion how often did you view your sitting time feedback on the app?		
> once/day	6/18%	–
Once/day	8/24%	–
Few times/week	8/24%	–
Once/week	1/3%	–
Infrequently	10/30%	–
In the past 6 months have you used the vibration function on the cushion?		
Yes	21/62%	6/46%
Used to but not anymore	9/27%	5/39%
No	4/12%	2/15%
How useful is the vibration function for reminding you to get out of your chair?		
5 (extremely useful)	11/48%	4/40%
4	9/39%	3/30%
3	1/4%	2/20%
2	1/4%	1/10%
1 (not at all useful)	1/4%	0/0%
How easy has the cushion been to use?		
Very easy	16/52%	9/69%
Easy	6/19%	3/23%
Neither easy nor difficult	7/23%	0/0%
Difficult	2/7%	1/8%
Very difficult	0/0%	0/0%
How obtrusive has the cushion been to your daily activities at work?		
Very obtrusive	2/6%	0/0%
Mildly obtrusive	7/21%	3/23%
Neither obtrusive nor unobtrusive	6/18%	0/0%
Mostly unobtrusive	5/15%	2/15%
Completely unobtrusive	13/39%	8/62%
The cushion has been useful for increasing my awareness of my sitting time at work		
Strongly agree	12/36%	4/31%
Agree	11/33%	4/31%
Neither agree nor disagree	8/24%	4/31%
Disagree	0/0%	0/0%
Strongly disagree	2/6%	1/8%
The cushion has encouraged me to reduce the time I spend sitting at work		
Strongly agree	10/30%	5/39%
Agree	12/36%	2/15%
Neither agree nor disagree	5/15%	4/31%
Disagree	3/9%	1/7%
Strongly disagree	3/9%	1/7%

Ratings were provided on a number of characteristics of the Darma cushion at 6 months and 12 months (Table 6). Data from the 32 participants using the cushion in the first 6 months suggested that it was easy to use (71% agreement), was not obtrusive (54%), increased awareness (69%), and encouraged less sitting (66%). The small sample using the Darma cushion at 12 months reported it as easy to use, largely unobtrusive and increased awareness, although only 54% agreed it decreased sitting (Table 5).

**Table 6:** Scoring on items concerning satisfaction with the Darma cushion reported at 6 and 12 months

	Comfort	Design/look	Battery life	Syncing data	Presentation of feedback	Navigation of feedback	Understanding feedback	Accuracy of assessing sitting
6-month data	3.50 (1.26)	3.75 (0.97)	3.39 (1.13)	2.96 (1.48)	3.26 (1.26)	3.26 (1.29)	3.22 (1.19)	3.26 (1.35)
12-month data	4.33 (1.00)	3.67 (1.00)	3.33 (1.12)	3.25 (1.28)	3.75 (0.89)	3.50 (0.76)	3.63 (0.92)	3.38 (1.19)

Ratings on a five-point scale (1 = low, 5 = high) reported as mean (standard deviation)

Focus group discussions on the topic suggested participants found other ways to set prompts, including using the Varidesk computer/phone app and Google Chrome Stand Up! Timer [level 2 sub-theme—prompts].

‘I’m using the computer prompt [Google Chrome Stand Up! Timer] now because I didn’t get on too well with the cushion’ (Intervention participant, focus group 6).

‘It helped a lot [Varidesk phone app], you could set the time, if you needed that regime at the start, you could say I am going to stand up for half an hour and then down again’ (Intervention participant, focus group 3).

At 6 months and 12 months, reasons given in the questionnaires for not using the cushion centred on lack of comfort, technological issues with the app and phone (e.g. syncing, storage and battery problems), length of charging lead and other reasons. The latter included a perception by some that it was not needed and that they could implement their own behaviour change without it. Similar comments were also made in the focus groups [level 3 sub-theme—cushion]:

‘It was very uncomfortable [and] it ran out of batteries so I never recharged it’ (Intervention participant, focus group 3).

‘The lead is really short, you had to plug it in, I think once it died’ (Intervention participant, focus group 3).

Some also reported that they used it initially but did not need it once the use of the height-adjustable workstation became more of a habit [level 2 sub-theme—factors that promote standing and habit formation]:

‘I did at the very start but then after that I actually found, because I was generally pretty good with my standing desk that I didn’t really see the requirement for the cushion’ (Intervention participant, focus group 7).

‘I think to start with, I had to use those timers and things to remind myself to stand up, but now it is just so natural … I just stand up until I feel like sitting down again or I stand up when I feel like I need to stand up’ (Intervention participant, focus group 3).

### Sitting time diary

Table 7 presents the quantitative responses to the diary. Most questionnaire respondents reported that they either never used or no longer used the diary to keep a record of their sitting (91%) nor used it for goal-setting (93%) within the first 6 months. Similar data were found at 12 months.

**Table 7:** Responses concerning the diary and goal setting

	6 months (*n*/%)	12 months (*n*/%)
Recording sitting/standing time in diary		
In the past 6 month(s) have you used the diary to keep a record of your sitting and/or standing time?		
Yes	5/9%	4/7%
Used to but not anymore	9/16%	9/17%
No	42/75%	41/76%
How often do/did you use the diary the record the time you spend sitting and/or standing?^a^		
Every day	5/31%	3/21%
Few times/week	6/38%	7/50%
Once/week	1/6%	0/0%
Infrequently	4/25%	4/29%
How useful is keeping a written record of your daily sitting and/or standing in helping you change your behaviour?^a^		
5 (extremely useful)	1/7%	1/8%
4	6/40%	4/31%
3	4/27%	3/23%
2	1/7%	1/8%
1 (not at all useful)	3/20%	4/31%
Goal setting element in diary		
In the past 6 months have you used the goal setting element in the diary?		
Yes	4/7%	4/7%
Used to but not anymore	19/33%	9/17%
No	35/60%	41/76%
How often do/did you use the goal setting element in the diary?^b^		
Every week	8/38%	5/39%
Every couple of weeks	8/38%	5/39%
Once a month or less	5/24%	3/23%
How useful is/was the goal setting in encouraging you to reduce your sitting time?^b^		
5 (extremely useful)	2/10%	1/8%
4	8/38%	4/31%
3	6/29%	6/46%
2	3/14%	1/8%
1 (not at all useful)	2/10%	1/8%

^a^Some people answered this question but did not answer the first question 'In the past 6 month(s) have you used the diary to keep a record of your sitting and/or standing time?' hence why the number of responses for this question is greater than the number of responses for the first question

^b^Only answered by those responding “Yes” or “Used to but not anymore” to the initial question “In the past 6 month(s) have you used the goal setting element in the diary?”

Reasons for not using the diary, including for goal-setting, were given in open-ended comments and included perceived lack of time and time pressure of their job (e.g. ‘work pressures—didn’t think about it’), forgetting, not finding it useful (e.g. ‘didn’t see point’, ‘doesn’t work for me’) and motivation (e.g. ‘effort of completing outweighs benefits’). Similar responses emerged from the focus groups (level 3 sub-theme—diary):

‘I think realistically you are probably not going to carry a paper diary around with you … it became another thing to either forget, like keep up with. And you always have your phone on you, so it’s easier just to write things on your phone’ (Intervention participant, focus group 3).

Some participants, however, stated in their questionnaire open-ended responses that they did not use the diary because they felt they did not need it. Some stated that their height-adjustable workstation was enough to encourage them to sit less, while others had created their own routine and habit (e.g. ‘stand when work allows me’, ‘I usually stand in the morning’).

### Feedback on sitting time

A large majority of the questionnaire respondents were in agreement, at both time points, that receiving feedback on their sitting time helped them think about their sitting, highlighted that they could be sitting too much, motivated them to change, helped plan and set goals, and was useful for reviewing progress (Table 8). Some of these findings were also discussed by focus group participants. [level 3 sub-theme—feedback].

‘You could see, in the information put in front of you, this is the chunk of your day sat down, you go ‘Oh my God’, and then you go ‘I need to make sure I stand up more’ (Intervention participant, focus group 4).

**Table 8:** Responses concerning receiving feedback from assessment of sitting time using the activPAL

Statement	6 months		12 months	
	Agree (*n*/%)	Strongly agree (*n*/%)	Agree (*n*/%)	Strongly agree (*n*/%)
Feedback on my sitting time made me think about how much I sit	21/40%	24/46%	28/55%	23/45%
Feedback on my sitting time highlighted to me that I sit too much	23/45%	19/37%	27/53%	15/29%
Feedback on my sitting time motivated me to make a change	22/42%	23/44%	24/47%	21/41%
Feedback on my sitting time helped me set goals around my sitting time and plan to change my sitting behaviour	20/39%	14/28%	22/43%	15/29%
Feedback on my sitting time was useful to review my progress	15/29%	30/59%	23/45%	27/53%

### Progress chats (i.e. coaching) with research team staff

All but one of the participants who were left in the study at 12 months had all four coaching sessions (*n* = 62). There were 72, 65, 65 and 63 participants participating in the first, second, third and final coaching sessions, respectively. At 12 months only, participants were asked in the questionnaire to reflect on the coaching and support provided by research staff through the progress chats that were provided (see Table 9). Participants reported that the chats helped them formulate plans (90%), helped them stay on track (90%), motivated them (94%), help them find solutions (87%) and provided support often enough (93%).

**Table 9:** Responses concerning the progress chats with research team (coaching sessions)

	12 months (*n*/%)
Chats with the research team have helped me formulate plans to sit less	
Strongly agree	18/33%
Agree	31/57%
No opinion	4/7%
Disagree	1/2%
Strongly disagree	0/0%
Chats with the research team help me stay on track with my plans to sit less	
Strongly agree	19/35%
Agree	30/55%
No opinion	1/2%
Disagree	4/7%
Strongly disagree	0/0%
Chats with the research team motivated me to sit less	
Strongly agree	24/44%
Agree	27/50%
No opinion	2/4%
Disagree	1/2%
Strongly disagree	0/0%
The research team would help me find solutions to barriers I have experienced to standing at work	
Strongly agree	20/38%
Agree	26/49%
No opinion	5/9%
Disagree	2/4%
Strongly disagree	0/0%
I felt the support from the research team was often enough throughout the intervention	
Strongly agree	29/54%
Agree	21/39%
No opinion	1/2%
Disagree	3/6%
Strongly disagree	0/0%

### Other lifestyle changes

The questionnaire results showed other lifestyle changes were made by 39% of intervention participants in the first 6 months. Of those reporting the nature of such changes, ten were positive (e.g. signing up for gym membership) and seven were negative (e.g. illness).

### Facilitators and barriers to behaviour change

Although the desk appeared to positively impact on behaviour change by providing participants the opportunity to stand whilst working, the lack of space on the Varidesk platform did appear to put participants off changing the desk position. This was mentioned by 50% of those reporting negative issues at 6 months and concerned papers and files falling off and a lack of space for handling multiple papers.

However, during the focus groups, many reported that this led to strategies to enhance the tidiness of their desk, thus creating a positive outcome [level 3 sub-theme—changes in work style].

‘My desk, for the first two or three days, it was slightly awkward, because I was working more narrowly. My arms weren't in the same places as they would have normally been in to operate the mouse. But very quickly, you got over that, and now I don't … well, within a week, I didn't even notice anything different about it. And it keeps everything very contained on your desk, and actually, encourages me to be a little bit more tidy, so that I can lift it and put it down without causing an avalanche’ (Intervention participant, focus group 1).

Other barriers to standing at both 6 and 12 months from the questionnaire data were musculoskeletal (e.g. ‘initial low back pain’, ‘initial leg pain’, ‘swollen ankles and feet’), ergonomic (e.g. ‘uncomfortable when typing a lot’, ‘sometimes couldn’t type when standing’, ‘wires would get caught’) and additional work issues (e.g. ‘remembering to use the workstation’, ‘change to established work pattern’, ‘feel awkward when standing’).

From the focus group findings, some participants commented on how the length of the intervention [level 3 sub-theme—length of intervention] gave them time to adjust to a different way of working [level 3 sub-theme—changes in work style] by incorporating standing (e.g. alternating from sitting to standing), which over time made them aware of the work benefits.

‘This has been quite a fundamental change, it’s made me think very differently about workspaces, environment, the way I interact with other people, you know … I’ve actually changed the way I work with my devices, and that means how I work with people, and the information and conversations I’m having, all that’s changed, so to make that [change] all in one go would be hard, and it’s needed a time period’ (Intervention participant, focus group 6).

‘But you soon adapt. You just remember when you pull it up [the desk] to move your paperwork in a bit, and put it in the right position’ (Intervention participant, focus group 2).

‘I concentrate better when I am standing up than when I am sitting down … If you have a difficult conversation on the phone, sometimes standing up is better because that way you are more confident as well’ (Intervention participant, focus group 2).

Regarding barriers to behaviour change, focus group participants felt that if they were having a stressful day or were engrossed in a certain task then they felt that they either needed to sit down or would simply forget to break up their sitting [level 2 sub-theme—aspects of the job].

‘I think it is hard to stand on stressful days. That’s what I found. You know, if there is a lot of stress in the office then it is difficult because you tend to sit. You’re fed up’ (Intervention participant, focus group 5).

‘Sometimes if you get involved in a bit of work or whatever, a few hours can go by like two or three and then you are like oh I have just been sat here for three hours, I haven’t even left my desk’ (Intervention participant, focus group 3).

The culture of sitting in meetings was highlighted as a barrier in the focus groups, with participants suggesting that managers should show support or roll out policy to stand more at work [level 1 main themes—wider/policy changes at work: barriers to behaviour change]. Participants did not feel comfortable or confident standing in meetings, particularly those meetings involving senior staff and/or when the manager does not recognise the importance of regularly standing.

‘Everyone has to be standing, otherwise the attention is on you, if you’re the one person standing in a meeting’ (Intervention participant, focus group 3).

‘I think manager buy-in is critical in this study. Because obviously if your manager is not supportive of you standing most of the time, then it can have a negative impact. And the interesting bit was if we [participant and manager] had meetings, we would have stand-up meetings. These tended to be much shorter and much focused as opposed to when you’re sat on a round table somewhere in a meeting room’ (Intervention participant, focus group 4).

‘But I think, yes, like you say, if you can get your manager to, yes, incorporate a ten-minute stand-up session in the middle, then that would be the best way to go’ (Intervention participant, focus group 4).

### Control participants

The coding of 41 open-ended questionnaire responses from 25 control participants who completed the questionnaire led to six main themes concerning lifestyle changes during the project: lifestyle changes (positive), lifestyle changes (negative), less physical activity, more physical activity, diet changes and other changes. Several said that they had moved house and this had caused a negative disruption to their lifestyle and health. Others reflected on positive lifestyle changes, including joining a weight management service. For physical activity, a significant number of comments reflected changes for both increasing and decreasing physical activity. A few stated positive efforts in changing their nutrition. Overall, a large number of life events were mentioned, and these appeared to have both positive and negative effects on lifestyle and health.

The second question asked whether being part of the study, despite being in the control group, had affected their sitting behaviour at home or work. Coding of 30 responses from 16 control participants led to four main themes: sit less, move more, change diet and awareness. The sit less theme reflected a number of changes control participants had made during the trial, including ‘I consciously get up from my desk frequently’, ‘make effort to stand more at certain tasks’ and ‘more aware at home—do not sit for too long’. Others reported an emphasis on moving more, reflecting sub-themes of using self-monitoring (e.g. ‘I count my steps daily’), incidental (e.g. ‘increased my stair use’) and exercise (e.g. ‘I try to walk at lunchtime’). Being part of the project seemed to create greater awareness in some control participants, mainly around sitting. This was reflected in a reduction in daily sitting time in control participants at 3-month follow-up, although not at further follow-up [8].

Finally, participants in the control group were asked whether any changes were made to their lifestyle after receiving health test results from the assessments. From 37 responses, 22 (60%) said that the tests did not have any impact on their lifestyle.

It became apparent from the focus groups that the feedback they received from the health measures at baseline and at 3-, 6- and 12-month follow-up was a key motivator to staying in the study [level 2 sub-theme—feedback from health measures].

‘It does make you more aware of, you know, the BMI and everything really … Its just to be aware of the whole, like, you’ve sort of had an MOT, haven’t you … Every four months you have one, which I think is good’ (Control participant, control focus group 2).

## Discussion

The process evaluation showed that participants had positive attitudes towards the height-adjustable workstation, with many using it on a daily basis. Most participants felt the education seminar increased their awareness of the health consequences of too much sitting and motivated them to change their behaviour. Receiving feedback on their sitting time and support from the research team also encouraged behaviour change. The Darma cushion and action planning/goal setting diary were seen to be less helpful for behaviour change. Several benefits were perceived by participants, including fewer aches and pains, improved cognitive functioning, increased productivity and more energy. Additionally, behaviour change seemed to be enhanced by behavioural feedback and regular contact with research staff through regular progress chats.

From this process evaluation, we can draw on the following categories recommended in Medical Research Council guidance [10]:

- intervention context: the contextual factors that might affect the implementation and outcomes of the intervention
- implementation: the implementation of the trial itself
- mechanisms of impact: any mechanisms helping to explain the impact of the trial.

### Context

There was a mix of positive and negative changes made during the lives of intervention and control group participants during the course of the trial. There was no apparent systematic bias in this regard. However, taking part in the study did appear to influence controls, at least in the short term. It is clear that any assumptions that control group participants remain stable in their behaviours during the trial are unfounded. In the present study, 40% of controls who responded felt that feedback from their assessments led to either confirmation of their situation, a greater awareness of issues, or actual changes to behaviour. Given that changes in primary and some secondary outcomes in the trial were largely in the desired direction [8], any differences seen in the trial outcomes between intervention and control participants may be an underestimation.

### Implementation

Five key implementation elements of the intervention assessed were the seminar and leaflet, the workstation, the Darma cushion, diary, and coaching progress chats. The seminar and leaflet achieved good reach; most read the leaflet and attended the seminar.

The height-adjustable workstation was implemented somewhat as planned, with participants given a choice of two designs (full electric or platform design) to accommodate different office set ups and preferences. However, the ordering and delivery of the workstations took longer than expected and this may have impacted on results at 3 months. However, nearly all participants used the workstations at least weekly, with about two-thirds using it daily. Fidelity of the Darma cushion and diary for self-monitoring and goal setting was moderate-to-low and very low, respectively. Participants engaged with the coaching chats and feedback from the activPAL device.

The Darma cushion was chosen based on feedback from participants in our development work [9]. However, in the intervention study, responses to the cushion were mixed in terms of its usefulness. Some participants sought out their own methods for receiving prompts to break up their sitting. This highlights that ‘one size does not fit all’ and future interventions may wish to consider flexibility in the tools offered to participants. It is likely that diaries for action planning and goal setting were considered too difficult and an extra task not worth doing. If greater use of the Darma cushion is to be encouraged, issues concerning comfort and enhanced technology are priorities to address. It is unlikely that one self-monitoring or prompting tool will satisfy everyone; therefore, there is a need to offer a greater choice of devices and tools for self-monitoring and prompting.

Comments from participants reflected low uptake of the diary and highlight that behaviour change techniques and other strategies provided by researchers may not always be seen in the same light by participants. Goal setting as a behaviour change technique will not be effective if adherence is low. This will more likely be the case for behaviour change techniques and tasks that require greater cognitive effort and time.

### Mechanisms of impact

The SMArT Work intervention was developed based on the Behaviour Change Wheel [9, 12]. A key element of this approach is the ‘COM-B’ framework where behaviour (B) is considered to be a function of the capability (C) of the individual, the opportunity (O) they have, and their motivation (M). These can be seen as mechanisms of behaviour change and are considered in this discussion.

The educational seminar and leaflet were well received. They appeared to increase awareness of the health consequences of too much sitting and provided motivation to make changes to the amount of time spent sitting. This addresses the motivation element of the COM-B framework and is more associated with ‘reflective’ forms of motivation, requiring participants to process information prior to decision making. In addition, the seminar and leaflet are likely to enhance perceptions of capability. One belief endorsed was ‘exercise may not offset the detrimental effects of prolonged sitting’. The belief that exercise does not offset the deleterious health effects of too much sitting is a controversial point in the contemporary literature and is probably a reflection of the development of the research field. Early epidemiological studies and meta-analyses suggested that higher levels of sedentary behaviour were associated with negative health outcomes when controlling for levels of moderate-to-vigorous or leisure-time physical activity (for example, [2, 13]). However, research has suggested that high levels of moderate-to-vigorous physical activity attenuate the effects of sitting on mortality [14, 15]. At the time of the development of the SMArT Work project, beliefs were more aligned with the comments emanating from the open-ended comments of participants. If we repeated the education, we would advise that the message reflect a more balanced view.

The height-adjustable workstation was also well received and was reported to have had numerous benefits. The two Varidesk models was viewed positively but some reported issues of a lack of space on the platform for papers. However, there was evidence that people adapted to this and it became a positive feature (i.e. they became tidier). The provision of such desks enhances participant’s capability and opportunity to reduce their sitting time.

Some of the qualitative findings support our quantitative results [6] concerning positive changes for musculoskeletal problems. Other process evaluations have also found participants reporting improvement in musculoskeletal issues [16]. Our qualitative findings also support our quantitative results around job performance, work engagement and recovery from occupational fatigue [8]. Importantly, most of the participants discussed how regularly standing benefited their work performance including concentration, confidence and creativity, and they also mentioned a positive impact on energy levels. These findings have also been reported in other qualitative studies evaluating small-scale height-adjustable workstations [16–18]. Specifically, Leavy and Jancey [18] found their participants reported that they felt height-adjustable workstations helped to create energy within work spaces and increased work performance.

The process evaluation also highlighted how standing at desks not only improved interaction between colleagues related to work tasks, but it also had a wider positive influence on engaging other employees not involved in the study in terms of reducing their sitting. Our intervention therefore provides new insights into how the development of social norms of regular standing has a widening influence on the workforce. Future trials could evaluate the reach of the effect of these types of interventions and assess changes in behaviour among non-participants.

Facilitators to changing sitting behaviours at work were explored during focus groups. It appeared that the most important components of the intervention to change sitting behaviour were the educational seminar and the provision of the height-adjustable workstations. The seminar was considered a strong influence in using the workstation and shows the importance of providing some education alongside the provision of height-adjustable workstations.

Very few barriers were reported by the intervention participants in adhering to the intervention. The ones that were reported included a lack of space on the height-adjustable desk platform that sits on top of an existing desk when raised to the standing position. However, participants often found ways to work around this during the intervention. The seminar session at the start of the intervention encouraged participants to identify other strategies in addition to using the desk to break up their sitting time. However, standing in meetings was considered difficult because of the wider predominant work culture of sitting and feeling self-conscious in the presence of senior staff. This is consistent with work by Mansfield et al. [19], and suggests that wider social behaviour change strategies are needed to make standing in meetings acceptable and the norm.

A large majority of the intervention participants reported very positively on their interaction with research staff, and especially for the progress chats (coaching) offered. These were reported as being helpful for motivation and planning, and appear to support the development of the processes in the COM-B framework, and in particular motivation and capability. Of note is that being part of the trial seemed to have positive consequences for just under half of the control group participants. These controls felt that they had made changes to their sitting behaviour, physical activity and nutrition.

In conclusion, the SMArT Work programme was successful in reducing sitting time for desk-based employees [8], and this process evaluation has provided valuable information on elements of the intervention and study that appear to have facilitated such behaviour change. These include the educational leaflet and seminar, the height-adjustable workstation, and behavioural feedback and interactions with research staff.

### Strengths and limitations of process evaluation

The main strengths of this process evaluation were the multiple methods used and two time points assessed through the questionnaires. A comprehensive set of indicators was assessed to judge context, implementation and impact of the intervention and randomised controlled trial. Limitations included the willingness of participants to respond fully to open-ended questions in the questionnaires. However, even though less than half the intervention participants took part in the focus groups, 84% of the clusters were represented. Not everyone completed the process evaluation questionnaires. Individuals taking part in the process evaluation could be biased. Taking part in the study did appear to influence the behaviour of the control group participants.

## Supplementary information

**Additional file 1.** Focus group guides.

## Data Availability

The datasets used and/or analysed during the current study are available from the corresponding author on reasonable request.

## Abbreviations

SMArT Work: Stand More At Work
NHS: National Health Service
