# Supplementary material for: Reducing sitting at work: process evaluation of the SMArT Work (Stand More At Work) intervention
Source: Trials. 2020 May 13;21:403. doi: 10.1186/s13063-020-04300-7 (PMC7218819; doi:10.1186/s13063-020-04300-7)
Supplement: Supplementary file 1 — Additional file 1. Focus group guides. [file 13063_2020_4300_MOESM1_ESM.docx]

SMArT Work: Stand More AT Work

FOCUS GROUP DEVELOPMENT

The focus group built on the 12-month process evaluation questionnaire findings. As the interviews were based on individual opinion, the focus group explored co-constructed responses underpinned by a realist-epistemological perspective [1]. The focus group gathered responses concerning the following: reasons for taking part in the study, impact of the height adjustable desk/platform on reducing sitting behaviour, barriers and facilitators to desk use, suggestions for changes to the intervention and suggestions for wider organisational implementation. A topic guide was used to facilitate the process of data collection, but this was used flexibly with scope for discussion of additional relevant topics that may arise.

PROCESS EVALUATION FG SCHEDULE (INTERVENTION)

- Why did you decide to take part in the SMArT Work study?
- Do you feel your manager was supportive of your participation in this study throughout the whole process? In what ways have they supported or not supported you?

The main aim of the study was to help office workers to reduce the time they spend sitting at work.

- Overall how do you feel the SMARTWORK intervention impacted on your sitting behaviour whilst at work?
- Would any of have made changes without this intervention?
- How have your colleagues impacted on your participation in the study?

Measurement sessions. We did a variety of different measures at each session and you received some feedback on your measures such as weight, body composition, waist circumference, blood pressure and cholesterol and HbA1c at baseline and 12 months.

- Did this information from the baseline measures impact on you in any way?
- How about the follow up measurement session?

The first component of the intervention you would have received was the seminar that presented an overview of sitting and health. The brief feedback that we received on this was good but I would like to understand a little more on how important the information was for you.

It’s important that our interventions are cost-effective and obviously face-to-face seminars increase cost.

- If we were to make this seminar an online training session do you think this would work for people? Would they complete it?
- Would it have the same impact as face-to-face? In what way is it different?
- Do you have any other suggestions on how we could get this sort of information out to people?

Tell me about your experiences of your height-adjustable desk.

- How did you incorporate it’s use in your working day?
- Have you incorporated standing more during your day outside of the desk use? How?
- Have your working patterns or work-load influenced how you have participated in this study?
- Can you tell me about any strategies you have used to use it? Did this change over time? Why?
- For those of you who it has helped to reduce sitting do you think you would have been able to reduce your sitting time at work without having the desk? If use has dwindled over time how might we encourage sustained use?
- It was apparent from our development work that knowledge about sitting behaviour and its impact on health was low. Did the information in the seminar impact on your use of the desk?
- For those of you who feel that the intervention has led to them making changes to their sitting behaviour at work, was there anything else that we have given you or you have sought for yourself that has been useful for helping you to make behaviour changes to your sitting?
- For those of you who feel you have made changes to your behaviour, have you experienced any benefits or negative impacts as a result e.g., how you feel, health, how you work etc
- For those of you who might feel that it hasn’t had an impact on your sitting behaviour, is there anything that we could have changed or added to this intervention that would have helped you?

Over the last year, we have tried to motivate a long term behaviour change, what we often find with these type of interventions is that people make changes initially, but these trail off over the longer term.

- Is there anything that we can add to the intervention to help people maintain any changes they may have made initially in reducing their sitting?
- Has the SMARTWORK intervention had any impact of your sitting and physical activity behaviour outside of work? In what ways?

Overall please can you sum up your experiences of the SMARTWORK intervention

If we were to deliver this intervention again, is there anything else that we could change or add to the intervention to make it more effective

PROCESS EVALUATION FG SCHEDULE (CONTROL)

1. How did you hear about the SMArT Work study?
2. What made you take part in the SMArT Work study?
3. Once you had read the information sheet, what were your expectations of the study? (*Prompt: Were you expecting to just be given a desk? What were your expectations of the other behavioural techniques mentioned in the PIS? What were you expecting for the measurement visits)*
4. Was your manager happy for you to take part in the study? Did you have to make time up for being absent from your desk for the measurement visits?
5. Was your manager supportive of your participation in this study throughout the whole process?
6. How did you find the process of the baseline measures? *(Prompt: comfort, length of time)*
7. Did the information you obtain from the baseline measures meet your requirements?
8. Did you find the information helpful?
9. Do you think upon receiving feedback on your health measures motivated you to change your behaviour?
10. How about the follow up measures? Was the information still of interest to you?

Reference:

[1] Braun V, Clarke V. Using thematic analysis in psychology. *Qualitative Research in Psychology* 2006;3(2):77-101. doi: 10.1191/1478088706qp063oa
